# Supplementary material for: IPEC-J2 rMdr1a, a New Cell Line with Functional Expression of Rat P-glycoprotein Encoded by Rat Mdr1a for Drug Screening Purposes
Source: Pharmaceutics. 2020 Jul 17;12(7):673. doi: 10.3390/pharmaceutics12070673 (PMC7408396; doi:10.3390/pharmaceutics12070673)
Supplement: Supplementary file 1 [file pharmaceutics-12-00673-s001.pdf]

pharmaceutics

MDPI

Article

Supplemntary Materials: IPEC-J2 rMdr1a and a New Cell Line with Functional Expression of Rat P-glycoprotein Encoded by Rat Mdr1a for Drug Screening Purposes

Lasse Saaby [1,*], Josefine Trasborg [2], Mikkel A. Rasmussen [1], Bjørn Holst [1] and Birger Brodin [2]

[1] Bioneer A/S, Kogle Alle 2, DK-2970 Hørsholm, Denmark
[2] Department of Pharmacy, University of Copenhagen, Universitetsparken 2, DK-2100 Copenhagen, Denmark
* Correspondence: lsa@bioneer-farma.dk; Tel.: +45-35336325

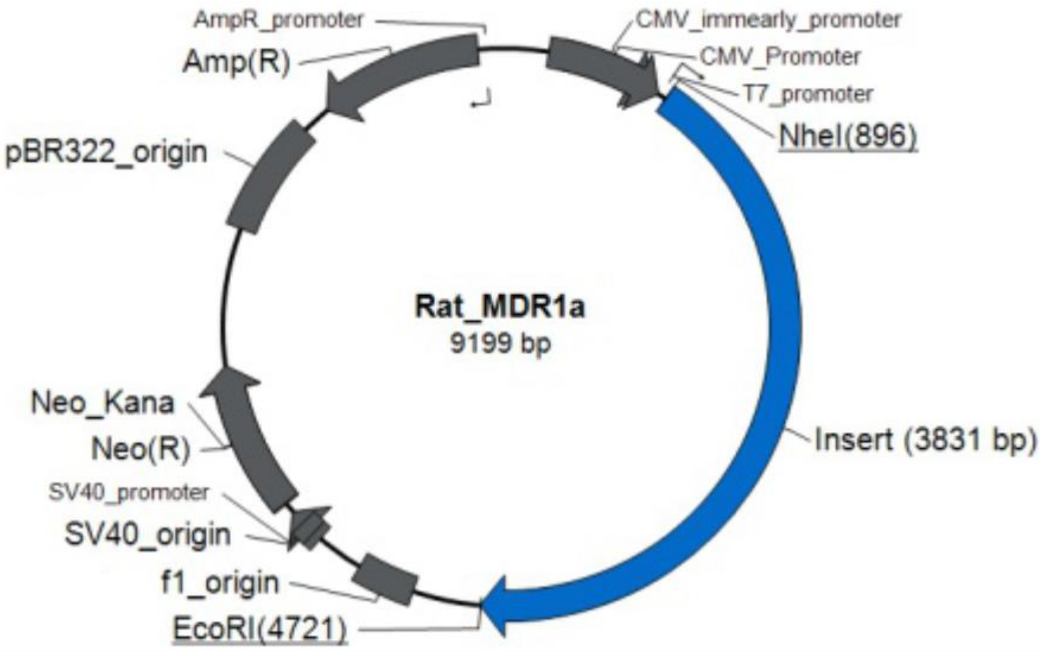

Figure S1. Graphical representation of the pcDNA 3.1(+) cloning vector encoding the rat Mdr1a gene.

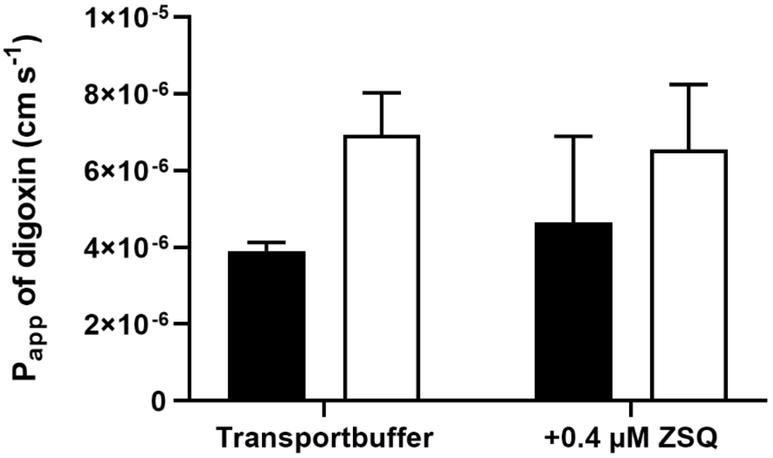

Figure S2. Bidirectional transport of digoxin across cell monolayers was measured in the presence or absence of the P-gp inhibitor, zosuquidar (ZSQ, 0.4 µM). Values are given as means ± SEM of 3 individual passages with 3 individual permeable supports for each passage and transport direction (n = 3, N_{total} = 9).

© 2020 by the authors. Submitted for possible open access publication under the terms and conditions of the Creative Commons Attribution (CC BY) license (http://creativecommons.org/licenses/by/4.0/).
